# A Non-Synonymous Point Mutation in a WD-40 Domain Repeat of EML5 Leads to Decreased Bovine Sperm Quality and Fertility

**DOI:** 10.3389/fcell.2022.872740

**Published:** 2022-04-05

**Authors:** Eriklis Nogueira, Filip Tirpák, Lauren E. Hamilton, Michal Zigo, Karl Kerns, Miriam Sutovsky, JaeWoo Kim, Dietrich Volkmann, Luca Jovine, Jeremy F. Taylor, Robert D. Schnabel, Peter Sutovsky

**Affiliations:** ^1^ Division of Animal Sciences, University of Missouri, Columbia, MO, United States; ^2^ Embrapa Pantanal, Corumbá, Brazil; ^3^ Programa de Pós-Graduação em Ciências Veterinárias, Universidade Federal de Mato Grosso do Sul, Campo Grande, Brazil; ^4^ AgroBioTech Research Centre, Slovak University of Agriculture, Nitra, Slovakia; ^5^ Department of Animal Science, Iowa State University, Ames, IA, United States; ^6^ Theriogenology Laboratory, School of Veterinary Medicine, University of Missouri, Columbia, MO, United States; ^7^ Department of Biosciences and Nutrition, Karolinska Institutet, Huddinge, Sweden; ^8^ Genetics Area Program, University of Missouri, Columbia, MO, United States; ^9^ Institute for Data Science and Informatics, University of Missouri, Columbia, MO, United States; ^10^ Department of Obstetrics, Gynecology and Women’s Health, University of Missouri, Columbia, MO, United States

**Keywords:** spermiogenesis, fertilization, acrosomal biogenesis, spermatozoa, SNP, fertility, bovine, knobbed acrosome

## Abstract

This study is part of a concerted effort to identify and phenotype rare, deleterious mutations that adversely affect sperm quality, or convey high developmental and fertility potential to embryos and ensuing progeny. A rare, homozygous mutation in *EML5* (*EML5* ^*R1654W*^), which encodes a microtubule-associated protein with high expression in testis and brain was identified in an Angus bull used extensively in artificial insemination (AI) for its outstanding progeny production traits. The bull’s fertility was low in cross-breeding timed AI (TAI) (Pregnancy/TAI = 25.2%; *n* = 222) and, in general, AI breeding to Nellore cows (41%; *n* = 822). A search of the 1,000 Bull Genomes Run9 database revealed an additional 74 heterozygous animals and 8 homozygous animals harboring this exact mutation across several different breeds (0.7% frequency within the 6,191 sequenced animals). Phenotypically, spermatozoa from the homozygous Angus bull displayed prominent piriform and tapered heads, and outwardly protruding knobbed acrosomes. Additionally, an increased retention of EML5 was also observed in the sperm head of both homozygous and heterozygous Angus bulls compared to wild-type animals. This non-synonymous point mutation is located within a WD40 signaling domain repeat of *EML5* and is predicted to be detrimental to overall protein function by genomic single nucleotide polymorphism (SNP) analysis and protein modeling. Future work will examine how this rare mutation affects field AI fertility and will characterize the role of EML5 in spermatogenesis.

## Introduction

Mutations which completely disrupt the function of a protein are termed loss-of-function (LOF). In essential genes located on autosomes (non-sex chromosomes), homozygosity for the same LOF mutation, or heterozygosity of two different LOF mutations (compound heterozygosity) results in lethality. Furthermore, autosomal mutations can be transmitted to progeny by either parent and, therefore, contribute to variation in genetic merit for both male and female fertility (Taylor et al., 2018a). The identification of fertility-affecting mutations can drive genetic progress in livestock industries and highlight genes that are important for human fertility, thus creating a role for large farm animals with sequenced genomes and extensive fertility records as important genomic and phenotypic biomedical models (Taylor et al., 2018b).

Interest in echinoderm microtubule-associating proteins (EMAPs) and EMAP-like proteins (referred to as EMLs or ELPs) was recently stimulated through the discovery of their involvement in neurological diseases (Sun et al., 2015) and human cancers (Nanjo et al., 2015; Wang et al., 2017). The first discovered EMAP, a 77-kDa protein that co-purified with microtubules and functionally facilitates microtubule dynamics, was discovered in sea urchin eggs (Suprenant et al., 1993; Hamill et al., 1998). Since this discovery, EMAP orthologs such as EMLs and ELPs have been found in taxa as diverse as echinoderms, eukaryotes, and vertebrates. Mammalian EMAP homologs (EML1-6) share structural features, including a conserved sequence in the N-terminus, termed the hydrophobic EML protein motif (HELP) and WD40 domains (Suprenant et al., 2000; Eichenmüller et al., 2001; Pollmann et al., 2006; Tegha-Dunghu et al., 2008). The crystal structure of the ∼70-kDa core HELP/WD40 region of human EML1 has been resolved, revealing a tandem atypical propeller structure, the TAPE domain, that is conserved within EML family members (Richards et al., 2014). The TAPE domain resembles the tandem ß-propeller structure of actin interacting protein 1 (AIP1) with the exception of one highly atypical blade built from two separate regions of the primary sequence (Voegtli et al., 2003; Mohri et al., 2004). While EML1-4 have been predicted to contain a single TAPE domain, EML5 and EML6 uniquely consist of multiple repeats of the complete archetypical EMAP protein and are phylogenetically more closely related to the original EMAP than to other EML family members (O’Connor et al., 2004; Fry et al., 2016).

EML5 contains 11 WD40 domain repeats and 3 HELP domains that are predicted to form three TAPE domains (O’Connor et al., 2004). The presence of an *α*-helical coiled-coil between the N-terminus and central regions of the protein also suggests that EML5 either homodimerizes or forms heterodimers with other EML family members, preferentially EML3 or EML4 (O’Connor et al., 2004). Furthermore, EML5 stands out as a unique family member within the EMLs for its lack of a calcium-binding EF-hand (O’Connor et al., 2004). Primarily expressed within the nervous system, EML5 was first detected in the rat brain, with the highest expression levels in the cerebellum, hippocampus, and olfactory bulb (O’Connor et al., 2004). However, in cattle, the Bgee database indicates that EML5 expression is highest in the reproductive system, adrenal gland, retina, and cerebellum (Bastian et al., 2021). Whilst the involvement of EMLs within mammalian reproductive systems has not been well characterized, research to date implicates several EML proteins in functional roles in both male and female gametes (Wang et al., 2015; Mańkowska et al., 2020; Yin et al., 2020). EML4 has been identified as a site of tyrosine phosphorylation during sperm capacitation (Wang et al., 2015), polymorphisms within *EML5* have been associated with decreased sperm motility (Mańkowska et al., 2020), and EML6 has been shown to participate in the regulation of oocyte meiotic division (Yin et al., 2020).

High expression of microtubule-associated proteins within the male reproductive system is not surprising as microtubule dynamics have large implications for sperm development in the testis, and have been shown to change in accordance with the developmental stage of sperm (Moreno and Schatten, 2000). Spermiogenesis, in particular, is highly dependent on microtubule dynamics to facilitate the developmental transformation of round spermatids into highly streamlined and differentiated spermatozoa. Spermiogenesis can be categorized into four distinct stages: Golgi, Cap, Elongation, and Maturation phases (Leblond and Clermont, 1952; Clermont and Leblond, 1955). At the onset of spermiogenesis, sperm development is centered on acrosome biogenesis, the formation of a large vesicular structure filled with proteolytic enzymes that localizes to the apical region of the sperm head. Effective acrosome formation is critical as the exocytosis of the acrosomal contents during fertilization helps to facilitate zona pellucida penetration and passage of the spermatozoon through oocyte vestments (Buffone et al., 2014). The initial stage of acrosome formation is driven by the Golgi apparatus, which produces glycoproteins and secretes coated proacrosomal vesicles from its *trans-*Golgi network (Tanii et al., 1998; Ventelä et al., 2000; Ramalho-Santos et al., 2001; Huang and Ho, 2006). These proacrosomal vesicles are then trafficked by microtubules and their associated proteins to their site of fusion on the apical aspect of the spermatid nucleus, forming the acrosomal granule (Schnapp et al., 1985; Sheetz et al., 1987; Tanii et al., 1998; Ventelä et al., 2000; Jordens et al., 2001; Ramalho-Santos et al., 2001; Dell, 2003; Huang and Ho, 2006). In the Cap phase of development, the acrosomal granule continues to be enriched and the structure begins to flatten and expand over the apical aspect of the nucleus. In the Elongation phase, the transient microtubular manchette forms and facilitates head shaping and intramanchette protein trafficking, while the expansion of the acrosome occurs over the apical aspect of the head and both the acrosome and chromatin undergo condensation. Elongation is also accompanied by a thinning of the cytoplasm (De Franca et al., 1995; Khawar et al., 2019). Finally, spermiogenesis is terminated by the Maturation phase; as the nucleus undergoes further condensation, the acrosomal granule expands over the whole acrosomal membrane and mitochondria localize on the midpiece of the newly formed sperm tail. Following acrosomal differentiation, the spermatid is deprived of its cytoplasmic droplet (Hess and De Franca, 2009; Guidi et al., 2018; Khawar et al., 2019). The intimate involvement of microtubule dynamics at all stage of spermiogenesis suggests that its dysregulation could result in major sperm morphological defects and sub- or complete infertility for the individual. Therefore, microtubule-associated proteins such as EMLs are of particular interest in investigations of sperm morphological defects and mutations that underlie variation in male fertility. In this study, we characterize a fertility-affecting, non-synonymous mutation found within a WD40 signaling domain repeat of EML5 (*EML5* ^R1654W^) in an Angus bull and investigate its involvement in sperm morphological defects and fertility outcomes.

## Materials and Methods

### Genomic Analysis and PCR Validation

An Angus bull was previously assessed by conventional and CASA sperm analysis (Nogueira et al., 2018) as well as by cross-breeding with Nellore cows *via* TAI and was shown to have reduced fertility. Protocol for TAI is described in our recently published studies (Nogueira et al., 2019; Rodrigues et al., 2019). This bull (UMC837) was previously sequenced (Hoff et al., 2017) and found to be homozygous for a predicted deleterious allele within the *EML5* gene. Using genotypes from the 1,000 Bull Genomes Project (Hayes and Daetwyler, 2019), we identified three additional Angus bulls heterozygous for this variant, and one homozygous for the reference allele to serve as a positive control (Supplementary Table S1).

Frozen semen from UMC837, UMC49060, UMC457, UMC3215, and UMC4517 was purchased from or donated by major purveyors of bull semen used in AI in the United States. All bulls were commercially distributed nationally and internationally at the time of this study and were considered to be fertile in AI service, albeit with varying levels of fertility. To validate the *EML5* variant, primers were designed using NCBI Primer-BLAST and the Bos-taurus_UMD_3.1.1 reference genome sequence (AC_000167.1). The bovine-specific *EML5* primers were synthesized by IDT (Coralville, IA, United States), and the primer sequences were forward 5’—AGG GTT TTA GAG CAA TGT GTG GT—3′ and reverse 5’—TGG CGA GGA CAG ACT AAG CA—3’. PCR was performed in a final volume of 10 µl [1 µl of gDNA (20 ng/μl), 1 µl of each primer (10 μM), 3 µl of GoTaq G2 Colorless Master Mix (Promega, Madison, WI, United States), 4 µl of nucleic acid-free water (Ambion, Austin, TX, United States)] using GeneAmp PCR System 9700 (Applied Biosystems, Waltham, MA, United States) for 30 cycles of denaturation at 94°C for 30 s, primer annealing at 60°C for 60 s and primer extension at 72°C for 30 s, and then the samples were incubated at 72°C for 5 min. The PCR products were confirmed for size by electrophoresis on 1% agarose gel with a 100 bp size marker (Promega, Madison, WI, United States). The PCR products were cleaned-up using a QIAquick PCR purification kit (QIAGEN, Hilden, Germany) and DNA concentrations were measured using a NanoDrop. The cleaned-up PCR products were Sanger sequenced at the University of Missouri DNA Core Facility for variant validation. For further comparison, a semen sample from an infertile, privately owned 100% stump tail syndrome bull was also obtained from the Theriogenology laboratory at the School of Veterinary Medicine, University of Missouri. The stump tail bull was genome-sequenced and found not to carry the *EML5* ^*R1654W*^ mutation.

### Antibodies and Reagents

All chemicals were purchased from Sigma-Aldrich (St. Louis, MO, United States) unless otherwise indicated. The main antibody used in this study was a rabbit polyclonal anti-EML5 (PA5-71086, Invitrogen—ThermoFisher Scientific, Rockford, IL, United States). A rabbit polyclonal anti-EML4 (ab86062, Abcam, Cambridge, MA, United States) antibody was also used. Secondary antibodies used in immunofluorescent analyses were tetramethylrhodamine-conjugated Goat anti-Rabbit IgG (GAR-TRITC; 81-6114, Zymed, San Francisco, CA, United States) and fluorescein isothiocyanate-conjugated Goat anti-Rabbit IgG (GAR-FITC; 65-6111, Invitrogen—ThermoFisher Scientific, Rockford, IL, United States). For acrosome integrity assessment, peanut agglutinin lectin (PNA) conjugated to Alexa Fluor 594 (PNA-AF594; L32459, Molecular Probes, Eugene, OR) and fluorescein isothiocyanate-conjugated PNA (PNA-FITC; Molecular Probes/Invitrogen, Eugene, OR) were used. 4′,6-Diamidino-2-Phenylindole Dilactate (DAPI; Molecular Probes/Invitrogen, Eugene, OR) was used as a nuclear DNA stain.

### Sperm Sample Processing

Frozen straws of bull semen from wild type, and *EML5* mutant bulls (both heterozygous and recessive homozygous) were thawed for 1 min in a 37°C water bath prior to further laboratory analyses. All semen samples were washed three times using warmed 4-(2-hydroxyethyl)-1-piperazineethanesulfonic acid (HEPES) buffered tyrode lactate medium (TL-HEPES, containing 10 mM Na-lactate, 0.2 mM Na-pyruvate, 2 mM NaHCO_3_, 2 mM CaCl_2_, 0.5 mM MgCl_2_; pH = 7.4, *t* = 37°C) supplied with 0.01% (w/v) polyvinylpyrrolidone (TL-HEPES-PVP) to remove the extender contained in the semen doses. All washes were performed by centrifugation at 350 *g* for 5 min using a swing-bucket rotor centrifuge. Samples were fixed in 2% (v/v) formaldehyde at room temperature (RT) for 40 min and further processing followed our established protocol (Sutovsky, 2004). Samples were stored in 200 μl of phosphate buffered saline (PBS) with 20 mM NaN_3_.

### Immunocytochemistry and Lectin Labeling

Sperm samples were placed into 0.1% (v/v) Triton-X-100 in PBS (PBST) on poly-lysine coated coverslips and incubated at RT for 40 min to allow both sperm attachment to poly-l-lysine and sperm permeabilization. Coverslips were washed and subsequently blocked in PBST with 5% (v/v) normal goat serum (NGS) for 25 min at RT. Primary antibodies, anti-EML5 or anti-EML4 were prepared in labeling buffer (1% NGS PBST) in ratios of 1:20 and 1:200, respectively. Spermatozoa were co-incubated with the primary antibody solution on the coverslips overnight at 4°C. The next morning, coverslips were submerged in 1% NGS PBST for 5 min. Coverslips were then co-incubated with secondary antibody GAR-TRITC (1:100), prepared in labeling solution along with PNA-FITC (1:400) and DAPI (1:100) for 40 min at RT in the dark. After co-incubation, coverslips were washed in 1% NGS PBST and mounted on glass slides with VectaShield mounting medium (Vector Laboratories, Burlingame, CA, United States). Coverslips were sealed with translucent nail polish to prevent drying. Images were recorded with a Nikon Eclipse 800 microscope (Nikon Instruments, Melville, NY, United States) equipped with a Retiga QI-R6 camera (Teledyne QImaging, Surrey, BC, Canada) operated by MetaMorph 7.10.2.240. software (Molecular Devices, San Jose, CA, United States).

### Immunohistochemistry

Bull testis tissues from an unrelated animal were obtained at a local slaughterhouse, fixed, paraffin-embedded, and sectioned using our standard protocol for tissue processing and antigen retrieval (Mao et al., 2018). Immunolabeling and imaging were performed with anti-EML5 (1:50) and anti-EML4 (1:1,000) antibodies. Secondary antibody solution contained GAR-TRITC (1:100), PNA-FITC (1:400), and DAPI (1:100) diluted in 1% NGS PBST. Images were recorded with a Nikon Eclipse 800 microscope with Retiga QI-R6 camera operated by MetaMorph 7.10.2.240 software.

### Image-Based Flow Cytometry

Approximately 10 million formaldehyde-fixed spermatozoa were permeabilized in PBST for 45 min at RT. Spermatozoa were subsequently blocked with 5% NGS PBST for 30 min at RT, and the anti-EML5 primary antibody (1:20 dilution) was added to sperm sample tubes and incubated overnight at 4°C. Negative controls included non-immune rabbit serum of comparable globulin concentrations as previously reported (Zigo et al., 2018). The following morning, spermatozoa were twice washed with 1% NGS PBST, and subsequently co-incubated with a species-specific secondary antibody such as goat anti-rabbit IgG (GAR-FITC), diluted 1:400 in 1% NGS PBST for 40 min at RT. Acrosome integrity was assessed using PNA-AF594, (1:2000 dilution), and DAPI (1:1,000 dilution) was used as a reference and nuclear contrast stain (Zigo et al., 2018). Both PNA-AF594 and DAPI were mixed and co-incubated with the secondary antibody. After incubation with secondary antibodies, spermatozoa were twice washed with 1% NGS PBST. Prior to performing flow cytometry, the sample aliquots were checked for fluorescence labeling under a Nikon Eclipse 800 epifluorescence microscope at ×20 and ×40 magnification. After positive labeling was confirmed, the final concentration was adjusted to 10 million spermatozoa per 50 μL through the use of a hemocytometer.

The fluorescently labeled samples were measured with an Amnis FlowSight imaging flow cytometer (EMD Millipore Corp., Seattle, WA, United States) fitted with a ×20 microscope objective (numerical aperture of 0.5) with an imaging rate of up to 2,000 events/sec. The sheath fluid was PBS, free of Ca^2+^ and Mg^2+^. The flow-core size was 10 μm diameter and speed was 66 mm/s, respectively. Raw images were acquired using INSPIRE^®^ software (Amnis-Millipore). The camera was set to 1.0 μm per pixel of the charged-coupled device. The image display dimension for the field of view was 60 and 8 µm depth of field. Samples were analyzed using four lasers simultaneously: a 405-nm line with intensity set to 10 mW; a 488-nm line with intensity set to 25 mW; a 642-nm line with intensity 20 mW and a 785-nm line (side scatter) with intensity set to 50 mW. A total of 10,000 events were collected per sample. Data analysis of the raw images was performed using the IDEAS^®^ software (Version 6.2.64.0; Amnis-Millipore), where the electronic images were compensated for channel crossover by using single-color controls (i.e., DAPI-only; AF-594-only; and FITC-only labeling of spermatozoa) that were merged to generate a multi-color matrix. The compensation matrix file was then applied to an experimental raw-image file (.rif), yielding a color-compensated image file (.cif). Displaying spermatozoa using Gradient RMS for the bright field channel allowed the gating of focused spermatozoa. Single-cell events were gated by combining the Area × Aspect Ratio scatter plot of the brightfield in the first step with that of PNA fluorescence, and the DAPI channel in the following step. A single cell population gate was used for the histogram display of mean pixel intensities by frequency for the following channels: AF594 (channel 2), DAPI (channel 7), and FITC (channel 11). Intensity histograms for the individual channels were then used to gate sub-populations with varying intensity levels and visual conformations. The intensity of DAPI (channel 7) was used for histogram normalization among different treatment groups. For a more detailed analysis, a scatter plot of each single gated object was drawn from the mean pixel intensities of AF594 (channel 2) vs. FITC (channel 11). Appropriate masks were applied to all relevant channels to exclude auto-fluorescent debris from feature calculation. The Feature Finder tool was utilized to identify the most relevant optical/morphometric feature of sub-populations when the mean pixel intensities were not sufficiently distinctive.

### Western Blotting

Bull semen straws from wild type and *EML5* mutant bulls (UMC837, UMC49060, and UMC457), were thawed in a water bath (37°C) and washed three times in warm TL-HEPES-PVA. After the final wash, sperm pellets were resuspended in lithium dodecyl sulfate (LDS) loading buffer [106 mM Tris HCl, 141 mM Tris Base, 2% (w/v) LDS, 10% (v/v) glycerol, 0.51 mM (0.75%) EDTA, 0.22 mM (0.075% w/v) Coomassie Blue G250, 0.175 mM (0.025% w/v) Phenol Red, pH = 8.5] supplemented with 2.5% (v/v) β-mercaptoethanol and protease and phosphatase inhibitor cocktail (Thermo Fisher Scientific). Approximately 100 mg of wildtype bovine brain tissue was used as an EML5 positive control and was homogenized using a prechilled mortar and a pestle before being mixed with reducing LDS buffer supplied with protease and phosphatase inhibitors. Spermatozoa and brain homogenates were left to incubate on ice for 30 min with periodic vortexing every 5 min. After incubation, suspensions were spun at 13,000 × g at 4°C. Extracts were transferred into fresh Eppendorf tubes and stored at −20°C until further use.

For polyacrylamide gel electrophoresis (PAGE), the NuPAGE^®^ electrophoresis system was used (Invitrogen, Carlsbad, CA, United States). Total protein equivalent of 15 million spermatozoa (∼30 µg) or 30 µg of cow brain extract (protein concentration estimated by BCA assay, cat #23227, ThermoFisher Scientific) were loaded per lane. PAGE was performed with NuPAGE™ 4-12% Bis-Tris gel (cat# NP0329BOX, Invitrogen, Carlsbad, CA, United States) using TRIS-MOPS SDS Running Buffer [50 mM Tris Base, 50 mM 3-(N-morpholino) propanesulfonic acid (MOPS), 0.1% SDS, 1 mM EDTA, pH = 7.7], with anode buffer and was supplemented with 5 mM sodium bisulfite to prevent the reoxidation of disulfide bonds. The molecular masses of separated proteins were estimated using Novex^®^ Sharp Pre-stained Protein Standard (cat # LC5800, Invitrogen, Carlsbad, CA, United States) run in parallel. PAGE was carried out at 80 V for 5 min to allow the samples to delve into the gel and then at 180 V for another 50 min. Power was limited to 20 W. After PAGE, proteins were electrotransferred onto a polyvinylidene difluoride (PVDF) Immobilon Transfer Membrane (Millipore Sigma, Burlington, MA, United States) using an Owl wet transfer system (Thermo Fisher Scientific) at 300 mA V for 1.5 h, using Bis-Tris-Bicine transfer buffer (25 mM Bis-Tris base, 25 mM Bicine, 1 mM EDTA, pH = 7.2) supplemented with 10% (v/v) Methanol, and 2.5 mM Sodium Bisulfite. After the electro-transfer, gels were stained with Coomassie Brilliant Blue (CBB) R-250 for protein load control.

The PVDF membrane with the transferred proteins was blocked with 10% (w/v) non-fat milk in tris buffered saline (TBS) with 0.05% (v/v) Tween 20 (TBST; Sigma-Aldrich) and incubated with the primary antibody against EML5 (PA5-71086, ThermoFisher Scientific, 1:1,500 dilution), overnight at 4°C. The next day, the membrane was incubated for 40 min with an appropriate species-specific secondary antibody, such as the HRP-conjugated goat anti-rabbit antibody (GAR-IgG-HRP, 1:10,000 dilution; cat # 31460 Invitrogen). The membrane was reacted with chemiluminescent substrate (Luminata Crescendo Western HRP Substrate; Millipore Sigma, Burlington, MA, United States) and the blot was screened with a ChemiDoc Touch Imaging System (Bio-Rad, Hercules, CA, United States), to record the protein bands. Afterward, the membrane was stripped with Restore™ Western Blot stripping buffer (cat # 21059, ThermoFisher Scientific) and re-probed with anti-β-tubulin antibody (1:4000, Developmental Studies Hybridoma Bank) followed by an anti-mouse secondary antibody (GAM-IgG-HRP, 1:10000 dilution; cat # 31430, Invitrogen) for protein load normalization purposes. The membrane was analyzed by Image Lab Touch Software (Bio-Rad, Hercules, CA, United States). Where not specified, procedures were carried out at RT. Membranes were stained with CBB R-250 after chemiluminescence detection for protein load control.

### Structure Modeling

HHpred analysis (Zimmermann et al., 2018) of the EML5 sequence identified Protein Database entry 4CI8, corresponding to the TAPE domain of EML1 (Richards et al., 2014), as a reliable template (E-value 1.6e-46; score 390.45) for modeling the C-terminal region of the protein (residues E1369-C1942) using MODELLER (Šali and Blundell, 1993). The same region of EML5 was also independently modeled with TrRosetta (Yang et al., 2020), followed by energy minimization and molecular dynamics refinement in YASARA (Krieger et al., 2009), as well as with AlphaFold2 (Jumper et al., 2021). The three methods produced similar models of the EML5 TAPE domain, whose N-terminal β-propellers [RMSD 1.30 Å (AlphaFold2/TrRosetta + YASARA, over 270 Cα)—2.59 Å (HHpred + Modeller/TrRosetta + YASARA, over 262 Cα)] expose R1654 at an essentially equivalent position at the end of their penultimate blade. The effect of the R1654W mutation on protein structure was modeled using the mutagenesis wizard of PyMOL (Schrödinger, LLC, New York, NY, United States), which was also used for model visualization and figure generation.

## Results

**FIGURE 1:**
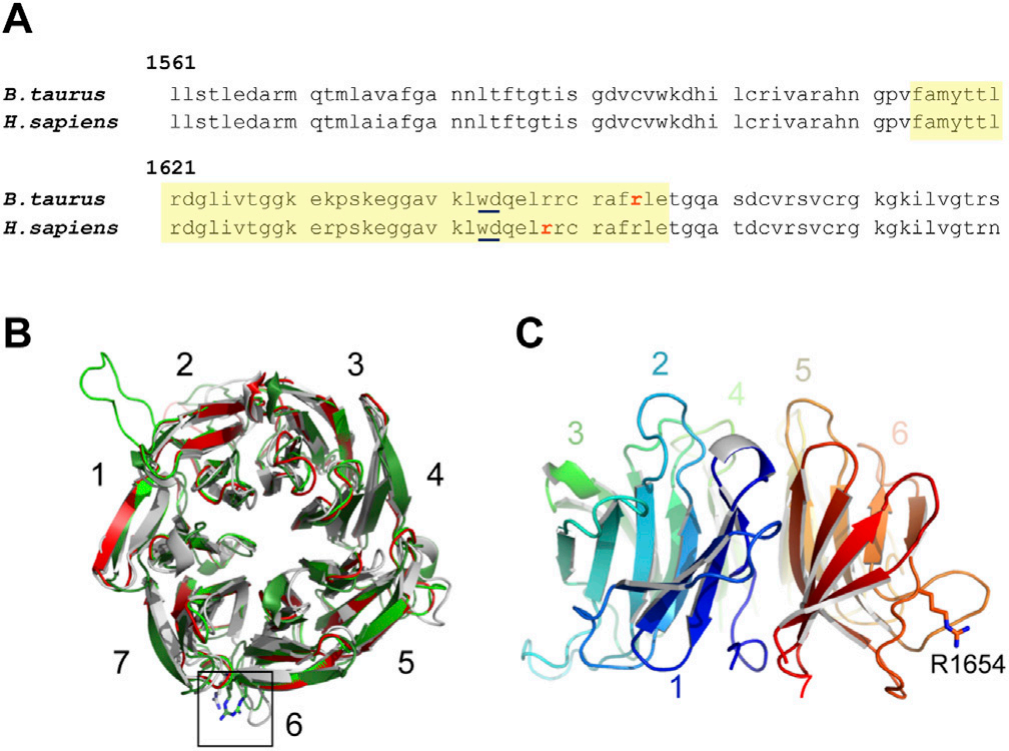
**(A)** Amino acid sequence of a WD40 domain repeat of EML5 in *B. taurus* and *H. sapiens*. The residues of the WD40 domain are highlighted in yellow. The affected arginine residue in bovine and the affected arginine residue predicted to cause cancer in humans are bolded in red. **(B)** Top view of models of the N-terminal β-propeller of the EML5 TAPE domain (residues G1378-S1698), generated by HHpred/MODELLER (light green), TrRosetta/YASARA (dark green), or AlphaFold2 (grey) and superimposed onto the crystal structure of the corresponding region of EML1 (red) (Richards et al., 2014). Numbers indicate the seven blades of the β-propeller, with a black box highlighting the predicted position of EML5 R1654, depicted in stick representation. **(C)** Side view of the AlphaFold2 model, rainbow-colored from the N-terminus (blue) to the C-terminus (red) to highlight the relative arrangement of the blades. Note how the side chain of R1654 is predicted to protrude into the solvent.

A rare SNP, predicted to be deleterious was discovered within a conserved WD40 domain repeat-encoding region (corresponding to protein residues 1614-1656) of the bovine autosomal (chromosome BTA10) *EML5* gene (echinoderm microtubule-associated protein-like 5 isoform X5, NCBI Reference Sequence XP_010807945.1) (*EML5* ^R1654^) (Figure 1A) in an Angus bull (UMC837) with low fertility in cross-breeding TAI (Pregnancy/TAI = 25.2%; *n* = 222) and general AI of Nellore cows (41%; *n* = 822) (Supplementary Table S2). All sequence data generated in this study have been deposited in the Short Read Archive (Supplementary Table S1). This variant is located at 10:100,310,158 on the ARS-UCD1.2 reference assembly (Rosen et al., 2020) and has the following genotype counts within the 1,000 Bull Genomes Run9 database: HOM REF G/G: 5,987; HET G/A: 74; HOM ALT A/A: 8 with an allele frequency of 0.7%, thus making the variant very rare. In the 1,000 Bull Genomes Run9 data, sample UMC837 (U.S.A) and an Australian Angus were the only Angus animals homozygous for the mutant allele, but there are an additional 31 heterozygous Angus animals from the U.S.A, Australia and New Zealand. The rare allele was not restricted to the Angus breed, as we further identified 4 homozygous and 21 heterozygous Normande animals. Additionally, heterozygotes were identified in the Ayrshire, Montbeliarde, Red Angus, Simmental, and Wagyu breeds.

At the protein level, this mutation results in a non-synonymous R-W substitution at amino acid residue 1654 (R1654W) and appears to be near-orthologous to the human R1648W mutation (NCBI accession 161436), associated with human cancer (Figure 1A) in the COSMIC Catalogue of Somatic Mutations in Cancer (Wellcome Sanger Institute). Using web-based predictors (PROVEAN, MetaSNP, PredictSNP, I-Mutant 2.0), the R-W substitution found in the homozygous EML5^R1654W^ bull was predicted to be detrimental or disease-causing and likely to decrease overall protein stability. Structural modeling of the C-terminal region of EML5, indicates that the substitution affects a residue within the WD40 β-propeller, which could have an impact on protein folding and/or interaction ability (Figures 1B,C).

**FIGURE 2:**
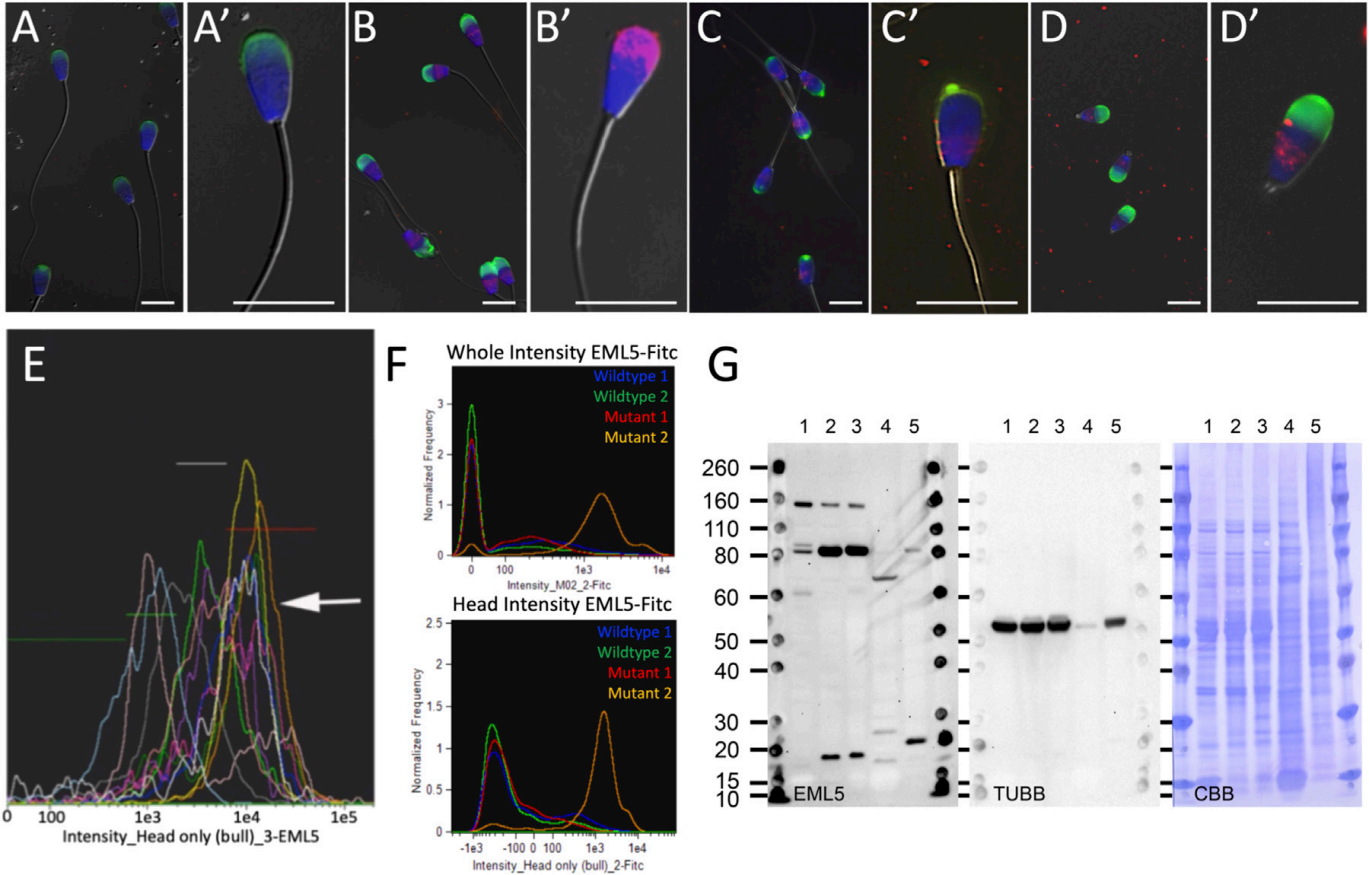
Immunolocalization of EML5 protein (red) in spermatozoa of wild type, heterozygous and homozygous *EML5* ^*R1654W*^ bulls. In all immunocytochemistry images acrosomes are stained with PNA (green) and nuclei are stained with DAPI (blue). Panel **(A,A′)** are typical labeling patterns of EML5 in the spermatozoa of wild-type bulls shown at two magnifications. Panel **(B)** shows EML5 labelling in a heterozygous EML5 ^*R1654W*^ bull (UMC49060) with the increased retention of EML5 in the piriform sperm heads highlighted in **(B’)**. Panel **(C)** shows the labelling of EML5 in the homozygous EML5^R1654W^ mutant bull (UMC837), with a single spermatozoon shown at increased magnification in **(C’)**. Images in **(D,D′)** show EML5 labelling in an asthenoteratozoospermic stump tail bull that has a suspected centriolar/microtubular defect but is wildtype for the EML5 mutation. Panel **(E)** is an image-based flow cytometric histogram overlap of EML5 fluorescence in bulls of varied but acceptable fertility. Each color/curve represents one sire. Intensities of EML5 induced fluorescence are gated from lowest to highest, left to right. The arrow points to the histogram of the heterozygous bull shown in panel **(B)**, with the highest median value of EML5-induced fluorescence. Panel **(F)** shows two image-based flow cytometric histograms showing EML5 fluorescence intensity in the whole cell and the isolated head region in two wildtype animals, one heterozygous EML5^R1654W^ bull (Mutant 1), and one homozygous EML5^R1654W^ bull (Mutant 2). Panel **(G)** is a Western blot comparing EML5 accumulation in the spermatozoa of wild type (WT) (1), heterozygous (2, UMC49060), and homozygous (3, UMC837) *EML5* ^*R1654W*^ mutants and a WT *EML5* bull who is an infertile asthenoteratozoospermic with 100% stump tails (4). Wild-type cow brain was used as a positive control (5). Additionally, tubulin (TUBB) and the total protein loading controls are also shown.

Immunodetection through indirect immunofluorescence (Figure 2) revealed an increased retention of EML5 protein within the sperm heads of the homozygous *EML5* ^*R1654W*^ bull (UMC837) that has a high incidence of piriform spermatozoa and knobbed acrosomes (Figures 2C,C′), as well as in the piriform sperm heads of a heterozygous *EML5* ^*R1654W*^ bull (UMC49060) (Figures 2B,B′). The increased retention of EML5 protein in the sperm heads of both heterozygous and homozygous carriers of the EML5^R1654W^ mutation was confirmed by image-based flow cytometry (Figures 2E,F). These findings were also validated using Western blotting (Figure 2G). Interestingly, the EML5 distribution and increased protein retention within the sperm heads were also seen in the spermatozoa of an unrelated, infertile asthenoteratozoospermic bull with 100% stump-tails, homozygous wild type for the *EML5* ^*R1654W*^ variant (Figures 2D,D′). This sample was used to examine whether the EML5 protein, a potential biomarker of sperm quality, accumulates in the sperm heads of infertile bulls with phenotypically well-defined sperm tail defects due to microtubule/centrosome disorders, regardless of *EML5* ^*R1654W*^ mutation status.

**FIGURE 3:**
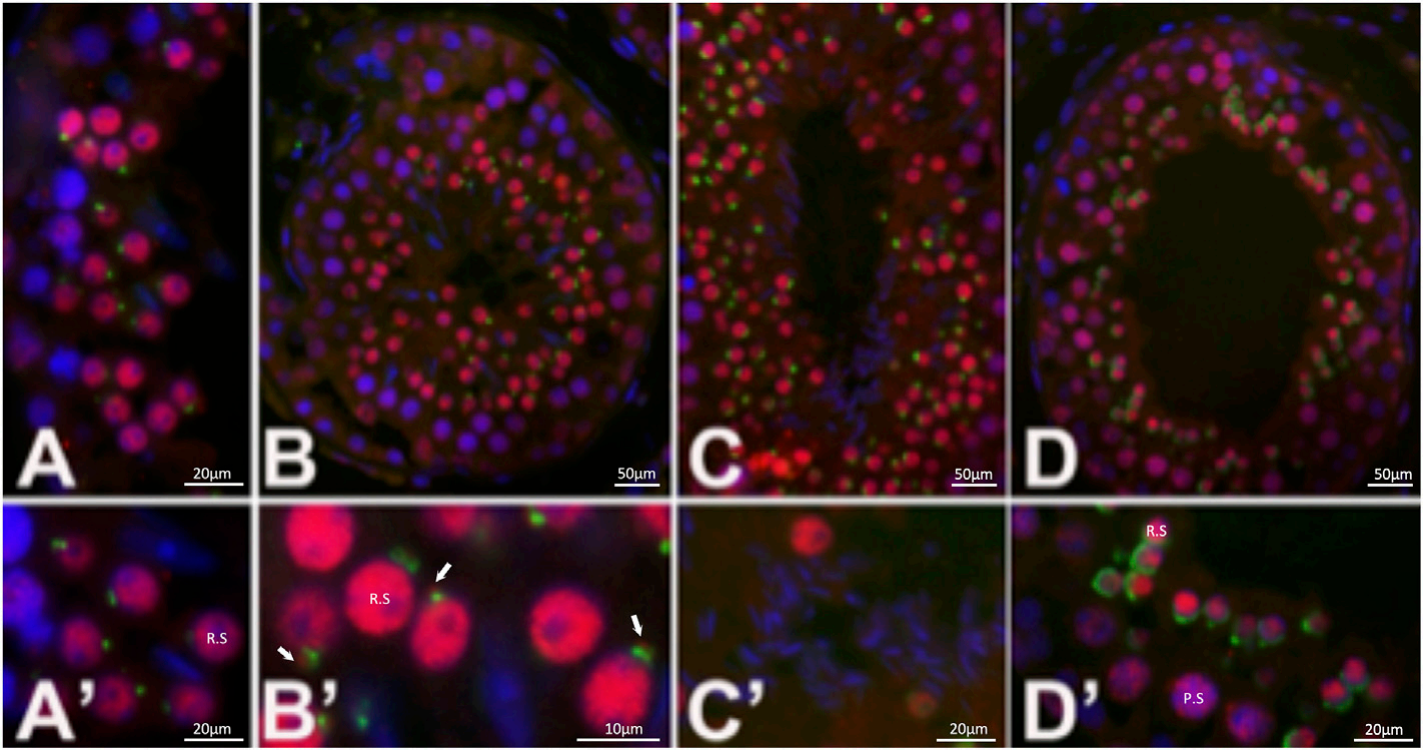
Distribution of EML5 (red) in bull testis. EML5 is detected in early step round spermatid (R.S.) nuclei **(A,A′,B,B′)** as well as in a distinct focus adjacent to the developing acrosomal granule **(B’)**; arrows; green, acrosomal labeling with lectin PNA. Nuclear labeling persists throughout spermatid elongation **(C,D)** and the formation of the acrosomal cap but weakens in late steps of elongation and sperm nuclear shaping **(C′)**. The earliest EML5 labeling was detectable in pachytene spermatocytes (P.S.) **(D′)**, where it is inter-dispersed between condensed chromosomes. DNA was counterstained with DAPI (blue).

**FIGURE 4:**
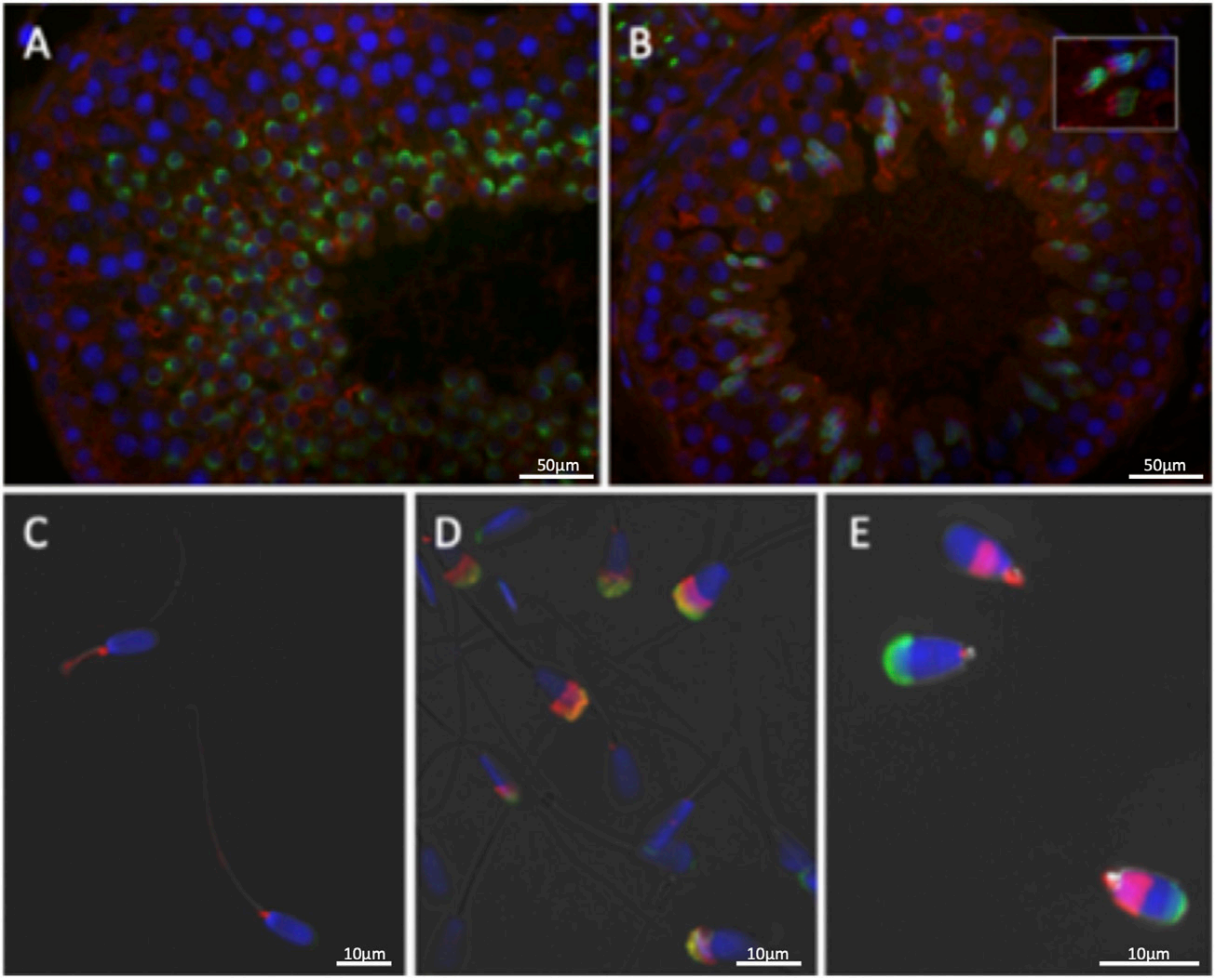
The distribution of EML5-related protein EML4 (red) in spermatozoa and testicular tissues is restricted to the cytoplasmic lobe of round spermatids **(A)** and the caudal manchette of elongating spermatids **(B)**. **(C–E)** Sperm EML4 distribution is altered by *EML5* genotype and sperm phenotype: Connecting piece and midpiece localization is prevalent in bulls with overall good sperm morphology **(C)**, while subacrosomal/acrosomal localization was found in the homozygous *EML5* mutant bull **(D)** and post-acrosomal sheath localization is prominent in an infertile asthenoteratozoospermic sire with 100% stump-tails **(E)**.

Immunohistochemistry of wildtype bovine testis sections (Figure 3) detected EML5 in the nuclei of early, round spermatids as well as in a distinct focus adjacent to the developing acrosomal granule (Figures 3A,B′). The nuclear localization persisted throughout spermatid elongation (Figures 3C,D) and the formation of the acrosomal cap, but weakened in the late steps of elongation and sperm nucleus shaping. The earliest EML5 labeling was detectable in pachytene spermatocytes, where it was interspersed between condensed chromosomes. The distribution of EML5-related protein EML4 (Figure 4) was also examined in wild-type and mutant spermatozoa and testicular tissues. It was found to be restricted to the cytoplasmic lobe of round spermatids (Figure 4A) and the caudal manchette of elongating spermatids in wildtype testicular sections (Figure 4B). Intriguingly, the distribution of EML4 was altered in the ejaculated spermatozoa of the *EML5* ^*R1654W*^ homozygous mutant bull as well as in the bull with stump-tail spermatozoa (Figures 4C–E). In wild type bulls with good overall sperm morphology, EML4 localized to the sperm tail connecting piece and midpiece (Figure 4C), whereas, in the homozygous *EML5* ^*R1654W*^ mutant bull, a subacrosomal/acrosomal localization was observed (Figure 4D). Furthermore, post-acrosomal sheath localization of EML4 was prominent in the spermatozoa of the aforementioned asthenoteratozoospermic bull with the stump-tail syndrome (Figure 4E).

## Discussion

This study characterizes the effects of a rare, deleterious mutation in the autosomal *EML5* gene, which encodes a microtubule-associated protein that is highly expressed in the testis and brain. We demonstrate that this mutant allele is present in many breeds world-wide. However, the very low allele frequency within these breeds supports the hypothesis of an old but deleterious mutation segregating in *Bos taurus* animals prior to breed formation, with the variant being maintained at low frequencies due to drift and only small selective disadvantages in heterozygotes. We identified a homozygous *EML5* mutant Angus bull used extensively in AI service, both in the USA and internationally, due to outstanding progeny production traits. The bull’s fertility was low in cross-breeding TAI (Pregnancy/TAI = 25.2%; *n* = 222) and in general AI to Nellore cows (41%; *n* = 822). Sperm morphological assessments identified prominent abnormal sperm phenotypes including piriform/tapered heads and knobbed or ruffled acrosomes, even within acceptable parameters. This striking phenotype of ruffling and inward/outward knobbing of the acrosome is difficult to detect using conventional low magnification bright field light microscopy, as used by breeding companies to assess semen quality of AI bulls, but is clearly discernible when utilizing acrosome staining in combination with immunofluorescence or image-based flow cytometry.

Confirmed by sequence analysis, this non-synonymous substitution affects an amino acid residue located within a WD40 domain repeat of the EML5 protein and corresponds to a human *EML5* mutation associated with cancer (Wellcome Sanger Institute). The WD40 domain repeat, also known as the WD or β-transducing repeat, is a short structural motif of approximately 40 amino acids, often terminating in a tryptophan-aspartic acid (W-D) dipeptide. Tandem copies of these repeats typically fold together to form a type of circular solenoid protein domain. WD40-repeat proteins are a large family found in all eukaryotes and are implicated in a variety of functions including but not limited to signal transduction, transcriptional regulation, cell cycle control, autophagy, and apoptosis (Xu and Min, 2011). The underlying functional commonality of all WD40-repeat proteins is coordinating multi-protein complex assemblies, where the repeating units serve as a rigid scaffold for protein interactions (Stirnimann et al., 2010). The specificity of these protein interactions is determined by the sequences outside of the repeats themselves. Examples of such complex forming proteins are G proteins (the β-subunit is a beta-propeller), the TAFII transcription factor, and E3 ubiquitin ligases.

In agreement with the present study, the negative effects of a SNP in *EML5* (SNP ID: *rs345056502*) have recently been reported in porcine by Mańkowska et al. (2020), who documented significant alterations in sperm motility and acrosome integrity along with increased lipid peroxidation. The bovine *EML5* ^*R1654W*^ mutation does not alter the protein product length but may cause changes to its ability to interact with other proteins or overall stability, by altering the conformation of a blade in the WD40 β-propeller or TAPE domain (Figures 1B,C). The accumulation of EML5 within mutant spermatozoa, as suggested by immunocytochemistry (ICC), and Western blotting may indicate that the mutant allele causes the protein to self-aggregate, or interact spuriously with other molecules. In both the homozygous and the heterozygous Angus bulls that were assessed, an increased retention of EML5 was observed in the sperm head, though small traces of EML5, likely a carryover from spermiogenesis, were also detected on occasion in the sperm heads of bulls that were homozygous wild type. Immunohistochemical investigations revealed that EML5 is primarily present in secondary spermatocytes and round spermatids, where its most likely function is the control of microtubule polymerization and severing during acrosomal vesicle assembly and manchette formation. Based on its nuclear presence in spermatocytes and spermatids, EML5 may also be involved in meiosis, spermatid nuclear shaping, and acrosomal vesicle biogenesis, with the latter function being supported by a high incidence of knobbed acrosomes in the homozygous *EML5* mutant. Furthermore, the increased prevalence of the outward knobbed acrosome phenotype was only present within the homozygous mutant bull, suggesting that one functional copy of *EML5* is sufficient for successful sperm development.

EML5 contains a putative dimerization domain, and likely homodimerizes or forms heterodimers with other EMLs, such as EML4 (O’Connor et al., 2004). EML4 localizes to the mitotic spindle and has been shown to promote microtubule stabilization. The N-terminus of EML4 preferentially binds to the C-terminus of α- and ß-tubulin where it is regulated by serine phosphorylation and facilitates proper mitotic spindle assembly. EML4 is currently receiving attention due to its possible fusion with anaplastic lymphoma kinase (ALK) forming pathological EML4-ALK variants associated with lung cancer (Chen et al., 2015; Sabir et al., 2017; Adib et al., 2019). However, EML4 is also strongly expressed in the testis, and here we show its distribution in testicular tissue and spermatozoa (Figure 4). EML4 is localized in the seminiferous epithelium with enhanced expression towards the lumen of the tubule, overlapping with the post-meiotic, haploid spermatids. Specifically, round spermatids express EML4 in the cytoplasmic lobe while in elongating spermatids EML4 is found on the caudal manchette. Spermatozoa of proper sperm morphology (WT bulls) have a prevalent localization of EML4 on the sperm tail connecting piece and midpiece, whereas a different EML4 distribution was detected in spermatozoa with aberrant morphology. Spermatozoa from the homozygous *EML5* ^*R1654W*^ mutant bull and an infertile bull with stump tails localized EML4 to the sperm head (acrosomal and post-acrosomal region, respectively). Different immunolabeling patterns suggest a possible interaction between EML5 and EML4 in developing spermatids, which could be disrupted by the amino acid substitution. Furthermore, the retention and/or change in localization of EMLs within mature spermatozoa may be indicative of microtubule dysfunction, as shown through our investigations using an asthenoteratozoospermic stump tail bull whose defects are suspected to stem from a centriolar or microtubule enucleation defect. Since the *EML5* gene is autosomal, with an independent assortment of the two copies of the carried chromosome 10 into gametes, the X/Y spermatid pairs in heterozygous bulls will be WT/WT, WT/Mut, Mut/WT, or Mut/Mut. Thus, three out of four spermatozoa produced by the heterozygous bull would have functional wild-type EML5 protein products, likely accounting for the much lower prevalence of the severe sperm phenotype within the ejaculate.

We conclude that the *EML5* ^*R1654W*^ mutation is associated with unique morphological and molecular phenotypes in bull spermatozoa. Homozygous and heterozygous *EML5* ^*R1654W*^ carrier bulls are rare within the AI industry but likely have compromised fertility. Whilst this may be tolerated by the AI industry due to their high genetic value for production traits, extensive use of *EML5* ^*R1654W*^ carrier animals could lead to an increased prevalence of spermatozoon morphological defects and decreased reproductive fitness within herds. Furthermore, the transmission of the mutation by the male gamete could contribute to paternal effects on pregnancy establishment, particularly if mated with female carriers of the mutant allele. Early pregnancy loss could be contributed by mutant EML5 protein expression within the preimplantation embryo, affecting microtubule-driven events during both interphase and mitosis phases of the cell cycle. Therefore, identification of fertility affecting mutations such as the *EML5* ^*R1654W*^ variant described here are important for cattle selection and reproductive management, and are significant for the development of human infertility diagnostics and therapies. The relevance of livestock models of human disease is also highlighted by the orthology between the *EML5* ^*R1654W*^ mutation and the WD40 mutation associated with human cancers. Future studies could investigate EML5 isoforms as potential cancer-testis antigens.

## Data Availability

The original contributions presented in the study are included in the article/Supplementary Material, further inquiries can be directed to the corresponding authors.
