# Supplementary material for: A Non-Synonymous Point Mutation in a WD-40 Domain Repeat of EML5 Leads to Decreased Bovine Sperm Quality and Fertility
Source: Front Cell Dev Biol. 2022 Apr 5;10:872740. doi: 10.3389/fcell.2022.872740 (PMC9037033; doi:10.3389/fcell.2022.872740)
Supplement: Supplementary file 1 [file DataSheet1.docx]

**Supplementary Table 1**: Carrier and wild-type Angus bulls used in this study, indicating their genotype at 10:100,310,158 and BioSample accession.

| Bull ID | Genotype | BioSample |
| --- | --- | --- |
| UMC837 | *A/A* | SAMN10940471 |
| UMC49060 | *G/A* | SAMN10940506 |
| UMC457 | *G/A* | SAMN10940450 |
| UMC32159 | *G/A* | SAMN10940503 |
| UMC4517 | *G/G* | SAMN05788497 |

**Supplementary Table 2:** Sperm concentration and motility parameters for the homozygous EML5^R1654W^ mutant bull were assessed during a previously published study (Nogueira et al., 2018) and are outlined below:

| Parameter | Value |
| --- | --- |
| Post Thaw Motility | 40% |
| Concentration of viable spermatozoa per straw | 9x10^6^/straw |
| CASA Motility | 35.6% |
| CASA Progressive Motility | 26.4% |
| Average Path Velocity (VAP) | 81.4 µm/s |
| Straight Velocity (VSL) | 70.72 µm/s |
| Linearity | 52.18 µm/s |
| Straightness | 84.98% |
| Amplitude of Lateral Head Displacement (ALH) | 6.17 µm |
